# To flag or not to flag: Identification of children and young people with learning disabilities in English hospitals

**DOI:** 10.1111/jar.12608

**Published:** 2019-05-16

**Authors:** Charlotte Kenten, Jo Wray, Faith Gibson, Jessica Russell, Irene Tuffrey‐Wijne, Kate Oulton

**Affiliations:** ^1^ Centre for Experience and Outcomes in Children's Health Illness and Disability (ORCHID), Great Ormond Street Hospital for Chidren NHS Foundation Trust London UK; ^2^ School of Health Sciences University of Surrey Guildford UK; ^3^ Faculty of Health, Social Care and Education Kingston University & St George's, University of London London UK

**Keywords:** children, hospitals, identification, learning disabilities, UK

## Abstract

**Background:**

Children and young people with learning disabilities experience poor health outcomes and lengthier hospital admissions than those without learning disabilities. No consistently applied, systematic approach exists across the NHS to identify and record this population. This paper describes practices in English hospitals to identify children and young people with learning disabilities.

**Method:**

*Interviews*: 65 NHS staff. *Questionnaire*: 2,261 NHS staff. Conducted across 24 NHS hospitals in England.

**Results:**

No standardized approach exists to identify children or young people with a learning disability or for this information to be consistently recorded, communicated to relevant parties within a hospital, Trust or across NHS services. Staff reported a reliance on parents to inform them about their child's needs but concerns about “flagging” patients might be a significant barrier.

**Discussion:**

Without an integrated systematic way across the NHS to identify children with learning disabilities, their individual needs will not be identified.

## 1 INTRODUCTION

Following on from the “Six Lives” report (Parliamentary & Health Service Ombudsman & Local Government Ombudsman, 2009), the current policy drive around the care of patients with learning disabilities (LD) (known internationally as intellectual disabilities) reinforces the view that to be in a position to meet needs and to prevent premature death, needs must first be identified. The UK's Confidential Inquiry into Premature Death of People with Learning Disabilities (CIPOLD), for example, recommends the “clear identification of people with LD on the National Health Service central registration system and in all health‐care record systems” (Heslop et al., 2014: 894). Identification, the process of “flagging,” to mark for attention or treatment in a specified way, has since been incorporated into the Care Quality Commission (CQC) best practice guidelines for children and young people with LD in hospital and in the recent LD improvement standards for NHS Trusts. Here, it states that “Trusts must have mechanisms to identify and flag patients with learning disabilities, autism or both from the point of admission through to discharge; and where appropriate, share this information as people move through departments and between services” (NHS Improvement, 2018: 6). There is limited guidance or evidence available as to what mechanisms of identification might be most effective or how they should be used in practice, but suggestions have included using “electronic flags in patient administration systems” (NHS mprovement, 2018: 6) and ensuring that flags are “clearly visible on the patient's records” (Glasper, 2017: 65). Fundamental to the process is that the flag is accompanied by “a statement of the reasonable adjustments required” (CIPOLD) such that care can be adapted (Sheehan et al., 2016) and monitored for adherence with equality legislation (Tuffrey‐Wijne et al., 2013).

There is little evidence to support what actually happens in practice. The identification of LD may not rest solely on a diagnosis as this does not necessarily indicate a need and the diagnostic category is not homogeneous. For some children and young people, a formal diagnosis of LD may never be made despite a continuing acknowledgement of their global development delay as they progress towards adulthood. Furthermore, where LD are known to exist, a better healthcare offer may arise, but there is limited evidence of that automatically leading to care that responds to or takes into account individuals’ needs. Evidence suggests that staff rely on parents to supervise, protect, advocate and look after their child's medical needs and behaviours whilst in hospital (Mimmo, Harrison, & Hinchcliff, 2018).

There is clearly an important difference between identifying that a child or young person has LD and recording what reasonable adjustments are required, and ensuring those reasonable adjustments are consistently delivered during their hospital admission in a timely way (Turner & Robinson, 2011). One study of adults with LD has found a lack of staff knowledge, expertise, willingness to identify and flag LD, and a reluctance to routinely record and “label” people exists across junior and senior staff (Tuffrey‐Wijne et al., 2013; Tuffrey‐Wijne & Hollins, 2014), and this reluctance can also be seen in other sectors such as education (Ho, 2004). Evidence is also lacking around staff education or training needs and their confidence and capability to meet the needs of this particular population.

“Pay More Attention” is a NIHR funded mixed methods study aiming to identify the factors that facilitate and prevent children and young people with long‐term conditions with and without LD from receiving equal access to high‐quality hospital care and services. This paper reports on the practices of a sample of English hospitals who employ (or not) a process to identify this population with LD. The wider context and overall findings are reported in Oulton et al. (2018).

## 2 METHOD

Health Research Authority approval was given for Phase 1 of the study (IRAS: 193932), the results of which are reported in this paper. Full ethical approval for this study was obtained from London‐Stanmore Research Ethics Committee (16/LO/0645). Staff who took part in interviews were provided with a participant information sheet and consent form and provided verbal consent prior to taking part in the interview. Survey participants were informed that their completion and return of the anonymized survey would be taken as their consent to participate.

Semi‐structured interviews were conducted with at least two senior managers per hospital with responsibility for the organization or management of care for patients with learning disability. These were conducted by CK or JR. Questions addressed the identification of children with LD and for all children: (a) equality of access to appointments, investigations and treatments; (b) the involvement of families as active partners; (c) satisfaction with care; and (d) safety concerns. Specifically, the interview explored what policies, procedures and systems were in place for identifying and/or flagging children within their Trust. The interview was piloted with two senior NHS managers.

An online questionnaire (with paper copies available) was developed for all clinical and non‐clinical staff with contact with this patient group to elicit perceptions of their ability to identify the needs of those with and without LD and their families and provide high‐quality care to effectively meet these needs. This was piloted with seven NHS staff at non‐participating sites with revisions made to improve clarity. Five questions related to the identification of children and young people with LD.

### 2.1 Setting

Twenty‐four NHS hospitals in England including specialist children's hospitals (*n* = 15) participated. The nine non‐specialist hospitals all had at least one paediatric inpatient ward as well as paediatric outpatient clinics and services. A local collaborator managed the study at each site.

### 2.2 Data management and analysis

#### 2.2.1 Qualitative

Audio‐recorded interviews were transcribed verbatim, anonymized and uploaded to NVivo 11 to utilize the framework option. Framework analysis, developed by Ritchie and Spencer (1994), was used to analyse the data. It is well recognized as an appropriate approach for applied research and focuses on describing and interpreting experiences in specific settings. It offers a systematic approach, which fitted the research and data collected (Ritchie & Spencer, 1994). The five stages of framework analysis were followed: familiarization with the data; identifying a thematic framework; indexing; charting and mapping; and interpretation (Ritchie & Lewis, 2003; Ritchie & Spencer, 1994). CK and JR charted, mapped and interpreted the data individually using Parkinson, Eatough, Holmes, Stapley, and Midgley (2015) as an example. The final review was undertaken by KO, JW and FG.

#### 2.2.2 Quantitative

Descriptive statistics were used to characterize the sample. Responses from participants at specialist children's hospitals were compared with those at non‐children's hospitals using Mann–Whitney tests in terms of (a) the perceived usefulness of identifying this population at an organizational level; (b) the usefulness of visibly flagging; (c) whether or not their organization provided information to identify these young people; and (d) their own confidence in identifying whether or not a patient in their care had LD.

## 3 RESULTS

### 3.1 Participants

The interview participants covered a range of senior staff within the participating hospitals with responsibility for people with learning disabilities. The present authors set out to conduct two interviews per site giving a minimum sample size of 48. This was to ensure all the interview questions were answered. The present authors continued interviewing staff until questions were answered with local collaborators asked to identify and invite additional participants. The final sample size was 65. Interviews lasted 30–45 min (Table 1).

**Table 1:** Participants in Phase 1 staff interviews and survey

Method	Number of hospital sites	Number of participants	Staff groups					
			Dr	N	AHP	LDN	SM	Other
Interviews	22	65	10	26	2	10	14	4

Method	Number of hospital sites	Number of participants	Staff groups				
			Dr	N	AHP	HCA	Non‐clinical/other
Survey	24	2,261	377	984	375	129	396

Abbreviations: AHP, allied health professional; Dr, doctor, HCA, healthcare assistant; LDN, learning disability nurse; N, nurse; SM, senior manager.

The quantitative and qualitative findings are presented using headings that provide a very broad linear narrative from identifying children with LD, what is done with that information, staff communicating with CYP with LD through to their own capacities and capabilities. This structure comes from the mapping and interpretation stage of framework analysis where patterns in the data were identified and offer a way to understand the current situation across the participating hospitals.

### 3.2 Identifying children with learning disabilities

Interview data revealed that 10 of the 24 hospitals in our sample had a process in place for identifying children and young people with LD and this was more prevalent in specialist children's hospitals (*n* = 8, 53%) than non‐specialist hospitals (*n* = 2, 22%). Staff referred to the use of “flagging” and “alerts,” although there appeared to be no clear distinction in the data between these two terms. Furthermore, no common or consistent formal or informal approach appeared to exist to identify this population with various practices being described including via a general practitioner (GP) referral letter or from a school, the use of hospital records; via pre‐assessment clinics, another hospital service or department; or via parents during the nursing assessment upon admission or in outpatients. It was evident that if a diagnosis of LD was made in a community setting, this information may or may not be transferred to the hospital. In four sites, staff felt that parents may not support the identification and subsequent labelling of children with LD because it felt simply wrong to “label” or there was a fear of getting it wrong even in Trusts where adults with LD are flagged. Interviewees tended to emphasize children as “individuals” and that all children should be treated “the same.” The implication was that if those with LD were flagged, it would mean these children were not being treated the same and this explained the hesitance or resistance to flag:

[W]e're a Trust with a specific Children's Hospital we'd like to say, ‘Well actually, all children matter and are individuals based on their presentation and it's not this blanket approach, if you like,’ it's that you, you know, dare I say it, sometimes get in a DGH [District General Hospital] or a lesser experienced work force who look after children, but we're all children's nurses in the Children's Hospital and Paediatricians so, absolutely the distinct needs are assessed and catered for. I guess regardless of whether they've got learning disabilities or not, you know, you could say a child who has, I don't know, chronic orthopaedic issues around brittle bones is catered for differently than a child who attends regularly with their diabetes, do you know what I mean? It's a very personalised approach we have here, I like to think as well. (Specialist Children's, Consultant)

In some hospitals, parental agreement was sought for their child with LD to be flagged with the benefits of flagging outlined to them.

Every family will be asked, ‘Do you consider your child or young person to have a learning disability?’ If they say ‘yes’, we'll talk them through what the flag does and the benefits of it and put the flag on. If they say ‘no’, we'll tell them what the benefits are and see if they want to change their mind [Where it is thought by clinical staff that a child does have a learning disability]. If they still say ‘no’, we won't force them to have that, but that does mean huge numbers of children who use our service are not flagged at the moment. So there is a flag, but it's not comprehensive in any way. (Specialist Children's, Deputy Chief Nurse)

This example highlights particularly well the challenges associated with identifying and flagging children and young people with LD in hospitals when no guidelines exist on how this should be done, by whom and on what basis.

Survey responses indicated that most staff saw the benefit of having a process in place, with 81% recognizing the *usefulness* of identifying children with LD at an organizational level and 72% recognizing the *usefulness* for such patients to be visibly “flagged” (72%) (Table 2). There were no differences between the specialist and non‐specialist hospitals in relation to such views. Only half of all survey respondents reported being given information about how to define LD, with an additional 14% not knowing whether they had information or not. The distribution of responses from specialist and non‐specialist hospitals was similar.

**Table 2:** Staff survey questions relating to flagging

Question	1	2	3	4	5
A. How useful do you think it is that children and young people with learning disabilities are identified at an organizational level (e.g., central database, electronic patient record)?	Extremely useful				Not at all useful
	1,014 (50%)	617 (31%)	269 (13%)	71 (4%)	39 (2%)
B. How useful do you think it is that children and young people with learning disabilities are visibly flagged at an organizational level (e.g., sticker on patient notes)?	Extremely useful				Not at all useful
	851 (43%)	572 (29%)	343 (17%)	130 (7%)	78 (4%)
C. In my role, I am routinely informed that a child or young person has a learning disability	Strongly agree				Strongly disagree
	396 (19%)	604 (29%)	560 (27%)	313 (15%)	190 (9%)
D. In your role, how easy is it for you to use these systems to identify that a child or young person has a learning disability?	Extremely easy				Not at all easy
	291 (18%)	472 (29%)	482 (29%)	213 (13%)	200 (12%)
E. How confident are you about identifying that a child or young person in your care/who you meet has a learning disability?	Extremely confident				Not at all confident
	562 (27%)	980 (47%)	379 (18%)	107 (5%)	47 (2%)

The present authors asked survey respondents about the systems in place at their Trust to identify and record that a CYP has a LD. Most frequently reported were medical notes (56%) or nursing notes (42%), followed by electronic documentation (27%). Seven per cent reported that no system was in place and 25% did not know what systems were in place at their Trust. Very few respondents referred to the use of databases (8%) or a sticker on the patient's notes (5%).

### 3.3 What happens when children with LD are identified?

When a child is known to have LD, the admission type, planned or unplanned, may affect what has been or can be put in place. Several sites reported in the interviews that where a child or young person was known to have LD or additional needs prior to admission, then a senior member of ward staff, for example, a ward manager would contact the child's parents and aim to identify and make reasonable adjustments for their admission. In contrast, unplanned admissions were regarded as more challenging for staff as reasonable adjustments could take time to organize despite the best efforts of the staff to accommodate a child's individual needs:

So when it's a planned elective case, we can, kind of, do a bit more planning. The difficulties, I think are it's harder when a child comes in acutely. Yes, they get the flag, but sometimes we aren't able to put everything in place that we can to try and support them in the best way. (Specialist Children's, Matron)

Once a young person with LD is identified, what happens with this information at each hospital varied considerably, from it simply being recorded in the child's hospital notes through to wider dissemination and engagement. Some hospitals had very clear formal pathways for sharing this information, for example, at one site, once a LD flag was added to a child's records, an automated email was sent to the LD nurse; at another site, an email alert was sent to the LD team for them to contact staff to review and consider reasonable adjustments or alternatively when a child was admitted with a LD flag already on their hospital notes an alert was sent to the LD team who then contacted relevant ward staff.

It's a mandatory field now, when you access somebody which has not been completed before, you have to say yes or no to whether or not they have a learning disability. If you click ‘Yes’ that then automatically emails our learning disability lead nurse who then puts a flag on the system, so anytime anybody accesses that patient on the system, it will come up with a flag, they can go in and see what their learning disability is. (Specialist Children's, Matron)

What we do with our alerts, is we've got something called [name of specific system] and our alert is linked to our email addresses and we all have an iPhone, so whenever that patient comes in, it's a live system, after two minutes of being clerked into the hospital, we'll get an alert on our phone to say the patient's name and where they are. (Non‐Specialist, LD nurse for Adults)

These pathways may be triggered through a flag or an alert already on the patient's records, identified during a pre‐admission appointment or recorded on their admission paperwork. Accessing specific information about a patient could be difficult; for example, staff may be able to see a child has LD but not all staff can access a child's record to ascertain more detail:

If people have got a learning disability and it's identified, they can have a flag put on, similar to those used in child protection flags or infection control flags. Then when you login to the system, you can see the patient's got a learning disability of some description, and then if you've got the right access level [permission level] and you go into that tab, you can see what their learning disability is. (Specialist Children's, Matron)

In contrast to formal routes, informal routes existed alongside or as standalone practices. Transfer of information between staff about children varied from a member of staff “knowing” the patient from a previous admission, verbal handover “huddles” during or when shifts changed, through to recording LD information in nursing and/or medical records.

Nearly half (48%) of all staff strongly agreed or agreed to being routinely informed that a child or young person in their care has LD (Table 2), with nearly a quarter (24%) of staff disagreeing or strongly disagreeing with that statement. With reference to particular staff groups, ancillary staff reported being routinely informed of patients with LD less than clinical staff (*X* ^2^ = 61.77; *p *< 0.001). Amongst clinical staff, nurses and healthcare assistants, but not doctors, were significantly more likely to report being routinely informed of a child/young person's LD than allied health professionals (*Z* = 2.74; *p *< 0.006).

### 3.4 Communicating about children with learning disabilities

It was acknowledged in the interviews that good communication between all parties was facilitative in identifying, understanding and meeting a young person's needs from pre‐admission to discharge. Staff felt that parents played a critical role in informing staff and a degree of responsibility lay with parents as repositories of information about their child, to share this information to “guide” healthcare professionals. However, this was not always reported as a seamless transition of information. Staff reported the presence of parental barriers, including linguistic barriers where English may not be a parent's first language; timely access to interpreters; where parents themselves may have LD; and practical barriers whereby parents do not bring the correct information about their child to the hospital or inhibit healthcare professionals from eliciting information directly from their child:

Barriers, in specific, if the parents are not willing to give that information or if they themselves have got some problems like, you know, safeguarding children; they might not have identified that their child has got learning difficulties. (Non‐specialist, Consultant)

[I]f I compare it with families where we know there's going to a barrier because of language, English isn't their first language, then it can be a real challenge to fit them into a ward round because you know you're going to have to use Language Line to communicate. So, I think it might put up artificial barriers. (Specialist Children's, Consultant)

Additionally, based on the assumption that a child had received a LD diagnosis, it was felt that some parents might be in denial about their child's LD, which could limit discussion about their needs and impact on implementing any reasonable adjustments:

So while staff may think this child has definitely got a learning disability, the family may not have quite got there yet, so may not want to say, ‘Yes, my child has got a learning disability.’ Flagging them is a bit of a label, isn't it? So there is a whole range of things which I think is why some staff are anxious about asking the question. (Specialist Children's, Deputy Chief Nurse)

They may fear their child will be stigmatized or that the presence of LD may lead to inequalities in their treatment, together resulting in parental reluctance to share information:

I think if their child's got very difficult behaviour or very, very hyperactive or things like that, I think they're a little bit worried about how their child is presenting and whether that will prevent things happening. I'm not necessarily saying it's real, but I think that sometimes it's a fear of the parents. (Specialist Children's, Learning Disability Nurse)

Additionally, issues associated with the degree of severity of a child's LD, with those at the milder end of the spectrum being potentially harder for staff to identify.

Unless it's overt or the parents actually formally identify, there may be milder learning difficulties that get missed because they're not specifically stated to the staff during the admission process, or pre‐admission. (Specialist Children's, Senior Nurse Manager)

It was noted that parents often had to repeat the same information to staff, and at times they may become frustrated with having to repeat information readily available in their child's hospital notes or hospital passport.

So, obviously, it's a real irritation for parents to have to go through the same information all the time, you know, at every contact. So we do have a system whereby, for those with particular issues, and they tend to be more medical issues, they can have an alert put on the system. (Specialist Children's, Consultant)

### 3.5 Staff capacities and capabilities

Overall 74% of respondents felt extremely confident or confident to identify a child or young person in their care or who they met has LD (Table 2 Q. E). Respondents from specialist children's hospitals reported feeling more confident to identify that a patient in their care had LD compared with respondents from non‐specialist hospitals (*Z* = 3.03; *p *= 0.002). However, in both specialist and non‐specialist hospitals, staff were more confident when their Trust gave them information about how to define LD (specialist hospital: *Z* = 7.40; *p *< 0.001; non‐specialist hospital: *Z* = 4.36; *p *< 0.001).

The data appear to show a correlation between Trusts that flag LD and staff views related to reasonable adjustments and safety. For example, staff from hospitals who flagged felt more able to identify what reasonable adjustments are needed for CYP with a long‐term condition and learning disabilities than those from hospitals who did not flag (*Z* = 4.37; *p *< 0.001), as well as feeling more confident that any reasonable adjustments would be accommodated in a timely way (*Z* = 2.62; *p *= 0.009).

Furthermore, staff from hospitals who flagged were more likely to report working in an environment that was deemed safe for meeting the needs of children and young people with a long‐term condition and learning disabilities than those from hospitals who did not flag (*Z* = 2.30; *p *= 0.021). The former were also more likely to report that they were always able to deliver safe care than those from hospitals who did not flag (*Z* = 2.68; *p *= 0.007) and felt more confident to safely manage challenging behaviour (*Z* = 3.07; *p *= 0.002).

The NHS groups staff into Pay Bands for non‐medical staff. Examples of different roles by Bands include Band 5 for a Staff Nurse and Band 6 for a Nurse Specialist through to Band 8 for a Modern matron. Survey data revealed that senior nursing and allied health staff (Bands 7 and 8) felt more able to identify children with LD (*Z* = −3.193; *p *= 0.001) but were not more likely to implement reasonable adjustments than their junior colleagues (Bands 1–4, 5 and 6) (*Z* = −1.707; *p *= 0.088). This finding was supported only in part by interview data, with interviewees suggesting that senior *and* more experienced ward staff were more likely to identify children with LD *and* to implement reasonable adjustments to meet their needs. Interviewees went on to describe a number of factors thought to impact whether CYP with LD are identified by staff and have their needs meet, from their initial and ongoing training, experience gained over time, having sufficient time with the patient and their family and access to appropriate resources. There was a general expectation by the interviewees that staff involved in a child's care would ask about any LD and/or would have read a patient's notes. However, it was recognized that staff may not have a sufficient level of knowledge about LD, which could mean this was omitted during admission; this was despite interviewees recognizing the value of specific training around caring for young people with LD to raise and maintain awareness and for training to be put into practice.

## 4 DISCUSSION

Reported here are a sample of strategies NHS English hospitals employ (or not) to identify children and young people with LD accessing their services, as well as staff views regarding the process. Prior to the introduction of the LD improvement standards for NHS Trusts (NHS Improvement, 2018), the present authors found that less than half of hospitals in our study had any process in place for flagging these patients and for those who did, there was no standardized way for this to happen or for this information to always be recorded, or for what is recorded to be confirmed as accurate, communicated to relevant parties within a hospital/Trust and acted upon.

A range of practices existed from a passive approach of simply noting a diagnosis of LD in a child's hospital records, asking parents’ permission for a flag to be applied through to active engagement with this information, including a formalized chain of events leading, ideally, to the implementation of reasonable adjustments to meet a child's needs. As a result, the patient experience between hospitals is likely to be very different.

During interviews, an emphasis was placed by staff on caring for children as individuals and as found in relation to the care of adults with LD (Tuffrey‐Wijne et al., 2013; Tuffrey‐Wijne & Hollins, 2014), there was a reluctance to label “difference” at an organizational or individual level that could be perceived to lead to discrimination. However, comparing the survey responses of staff working in hospitals that flag LD with those working hospitals that do not revealed that the opposite occurs in practice, strengthening the argument for those with LD to be identified on the grounds of increased safety and to achieve equity of treatment and individualized care (Oulton et al., 2018) through identifying the need associated with that LD (Tuffrey‐Wijne & Hollins, 2014) and accommodating those needs through the provision of reasonable adjustments.

Interviewees raised a number of issues about the complexities of identifying children and young people with LD, in relation to their parents. They felt that parents may not support a process of “labelling” their child, they may not agree with the diagnosis or they may need support themselves to articulate their views due to their own LD or language barrier. Such findings highlight the need for clear guidelines about how parents should be involved in the process of flagging children and young people with LD in hospital, particularly as some of the staff interviewed felt that children with “mild LD” can go unrecognized. In adult services it has been reported that “those without a formal diagnosis may remain invisible” (Sheehan et al., 2016: 6). Included in these guidelines must be information about the use of definitions around LD, as well as how to ask parents the relevant questions in a sensitive manner.

Less than half of staff who the present authors surveyed reported being routinely informed that a child in their care has LD, but at least three quarters of them reported seeing the benefit of formally identifying this population and feeling confident to do this. Such findings suggest that the primary issue is the communication of information between staff rather than a lack of staff knowledge or willingness to identify the population, as found in adult services (Tuffrey‐Wijne et al., 2013). The present authors cannot know how well feelings of confidence correlate to the ability to correctly identify children and young people with LD, but the present authors do know that staff who have been given information about how to define LD report feel more confident to do so in practice and that perceived confidence is greater for senior staff and for staff working in specialist children's hospital. Despite such confidence, there was a perception from interviewees that a degree of responsibility fell to parents to inform staff about their child's needs, supporting the argument that a flag alone is not sufficient, but must be accompanied by “a statement of the reasonable adjustments required” for each child. Whilst parents are an expected source of information about their child, a reliance on them may mask a need for staff to be better educated about LD. Certainly, published evidence from this study states that staff do feel less capable to meet the needs of children and young people with LD compared to those without LD and the former are perceived by staff to be less safe than those without LD (Oulton et al., 2018). Further research needs to identify the underlying causes, including whether there is a case for increasing the quantity and quality of undergraduate training around LD. This would mean that individuals’ knowledge is not dependent upon their seniority or length of experience, their place of work or the parent's willingness or ability to proactively engage with them about their child's needs. However, as recognized by the LD improvement standards for NHS Trusts, Standard 3 requires training *within* hospitals must be responsive to patterns of local need (NHS Improvement, 2018). Being better informed and trained should lead to increased confidence and a more equitable partnership between parents and staff when discussing and providing care for children with LD.

### 4.1 Limitations

The data presented here provide the perspective of NHS hospital staff. It is important to gain the views of children, young people and their parents to understand how LD is correctly identified (or where identification is missed or an incorrect “label” applied) and the value of having LD identified and the perceived implications that this information will have on practice.

This wider study from which this data comes sought to map the provision of care for children with long‐term conditions, particularly those with LD through a mixed methods approach. The NHS staff who participated in interviews were identified locally and comprised a wide variety of roles (e.g., medical consultants, matrons, clinical nurse specialist and managers) which may have produced a lack of consistency in the data. At two sites (non‐specialist hospitals), no staff agreed to be interviewed. The 30‐min interview was designed to encourage participation but limited in‐depth discussion around site practices. Additionally, where a difference by “pay band” has been identified, the present authors cannot be certain that a lower band indicates a lack of experience in working with children and young people with LD.

Although the study did not aim to be proportionally representative of specialist children's hospitals and non‐specialist hospitals, children are more likely to receive treatment in their local hospital, which for many, is unlikely to be a specialist hospital for children. If this study was repeated, a sample that better reflects this form of access might be incorporated.

## 5 CONCLUSION

There is no standardized way of identifying children and young people with LD and their individual needs across hospitals in the NHS in England. The identification of these children needs to be consistent within and across the NHS and for this identification to be the beginning of a standardized process whether through flagging or another form of alert. This is essential to not only inform health and social care professionals that a child has a LD but what reasonable adjustments they require as individuals and to ensure that these are put in place to provide them with the best possible care. This is dependent on healthcare professionals having a level of training, confidence, skills and knowledge to actively and positively respond to initially identifying LD and being able to put into place the adjustment required, drawing on the available resources from the individual to the institutional. A robust and comprehensive system that works across the whole NHS is required to identify this population and their needs and provide equity of access to resources to meet these needs.

## CONFLICT OF INTEREST

No conflict of interest has been declared.
